# Maternal Tetanus Toxoid Vaccination and Neonatal Mortality in Rural North India

**DOI:** 10.1371/journal.pone.0048891

**Published:** 2012-11-09

**Authors:** Abhishek Singh, Saseendran Pallikadavath, Reuben Ogollah, William Stones

**Affiliations:** 1 Global Health & Social Care Unit, School of Health Sciences & Social Work, University of Portsmouth, Portsmouth, United Kingdom; 2 Department of Obstetrics & Gynaecology, Aga Khan University, Nairobi, Kenya; Public Health Ontario, Canada

## Abstract

**Objectives:**

Preventable neonatal mortality due to tetanus infection remains common. We aimed to examine antenatal vaccination impact in a context of continuing high neonatal mortality in rural northern India.

**Methods and Findings:**

Using the third round of the Indian National Family Health Survey (NFHS) 2005–06, mortality of most recent singleton births was analysed in discrete-time logistic model with maternal tetanus vaccination, together with antenatal care utilisation and supplementation with iron and folic acid. 59% of mothers reported receiving antenatal care, 48% reported receiving iron and folic acid supplementation and 68% reported receiving two or more doses of tetanus toxoid (TT) vaccination. The odds of all-cause neonatal death were reduced following one or more antenatal dose of TT with odds ratios (OR) of 0.46 (95% CI 0.26 to 0.78) after one dose and 0.45 (95% CI 0.31 to 0.66) after two or more doses. Reported utilisation of antenatal care and iron-folic acid supplementation did not influence neonatal mortality. In the statistical model, 16% (95% CI 5% to 27%) of neonatal deaths could be attributed to a lack of at least two doses of TT vaccination during pregnancy, representing an estimated 78,632 neonatal deaths in absolute terms.

**Conclusions:**

Substantial gains in newborn survival could be achieved in rural North India through increased coverage of antenatal TT vaccination. The apparent substantial protective effect of a single antenatal dose of TT requires further study. It may reflect greater population vaccination coverage and indicates that health programming should prioritise universal antenatal coverage with at least one dose.

## Introduction

Neonatal tetanus is an important preventable cause of neonatal mortality. Estimates from the year 2000 of the distribution of direct causes of death indicate that the infection accounts for 7% of neonatal deaths worldwide [1]. Despite a considerable global mortality decline over the last two decades, continued high incidence in some countries results in a continued high burden of deaths, with an estimated 130,000 newborn deaths in the year 2004 from neonatal tetanus [2]. Most of these deaths occur in a limited number of populous countries such as India and Nigeria [3], [4] that have not yet managed to assure comprehensive health cover especially to rural communities. In the South East Asia Region, WHO has attributed 4% of the neonatal deaths to neonatal tetanus [5]. A recent systematic review estimated that neonatal tetanus contributed to between zero and 21% of the all neonatal deaths in India [6].

In India, a recent study of newborn infections projected to the population level indicated that neonatal infections alone accounted for 270,000 (250,000 to 290,000) neonatal deaths [7]. Within the country however the main mortality burden is concentrated in the northern states [8]. Estimates from 2008 suggest that 56% of deaths in India due to neonatal tetanus occurred in the northern states of Rajasthan, Uttarakhand, Uttar Pradesh, Madhya Pradesh, Chattisgarh, and Jharkhand. Of these the state of Uttar Pradesh alone contributed 28% of the deaths and Rajasthan 16% [9]. Strikingly, the relative contribution of the northern states to India's tetanus mortality burden has tripled during the last two decades, rising from 18% in 1999 to 56% in 2008 [9], [10]. Two studies from northern India give widely differing estimates of the burden of neonatal tetanus, from four to 38% of the total [11], [12]. As these are the states that bear the maximum burden of infant and child mortality in India [13], understanding the actual contribution of neonatal tetanus to overall mortality and any associations is a public health priority.

As case fatality rates for neonatal tetanus are very high the programmatic emphasis has been on prevention through hygienic birth practices but most importantly by a strategy of antenatal vaccination to provide protective levels of immunity to the newborn [14]–[16]. Vaccinating mothers for tetanus during pregnancy is likely to have maximal impact in communities where neonatal tetanus is common and where significant proportion of deliveries takes place in unhygienic conditions and mostly attended by untrained professionals [15]. These are typical of conditions in rural north India where a pattern of inequalities in maternal health care provision is seen [17].

While the tetanus toxoid vaccination of pregnant women was included in the WHO's Expanded Program on Immunization (EPI) as early as the mid-1970s and is now a standard practice [18], the evidence base to support the mortality effect is extremely limited. A recent systematic review yielded only one randomized controlled trial and one well-controlled cohort study. Immunization of pregnant women or women of childbearing age with two doses of tetanus toxoid was estimated to reduce mortality from neonatal tetanus by 94% [3]. However, the main limitation identified in the review that could influence the resulting effect estimate was the dearth of high-quality trials. This systematic review also included the two studies listed in the Cochrane review (‘Vaccines for women to prevent neonatal tetanus’), one from Columbia in 1966 and the second from Bangladesh in 1980 [19]. The Bangladesh trial was originally designed to test a cholera vaccine and tetanus toxoid was given to participants in the control group [20]. Hence the Bangladesh trial is likely to substantially underestimate the effect on neonatal tetanus mortality [3], [19]. The Columbia trial had a third arm containing participants that refused vaccination and that data from this arm were not included in the analysis [19], [21]. For India, two small-scale studies examined the effect of immunization of pregnant women with tetanus toxoid on neonatal mortality [22], [23].

We aimed to examine antenatal vaccination impact in a context of continuing high neonatal mortality in rural northern India using data from a statistically generalisable population sample survey.

## Data and Methods

### Ethics statement

This study used anonymised survey data made available for academic use, for which ethical approval was not required.

### Data

The present study uses data from the third round of the Indian National Family Health Survey (NFHS) conducted during 2005–2006 (NFHS 2005–06). NFHS is a nationally representative, large scale, repeated cross sectional household survey. The principal objective of NFHS is to provide state and national level estimates on fertility, mortality, and family planning. The previous two rounds of NFHS were conducted during 1992–93 and 1998–99. The survey adopted a two-stage sample design in most rural areas and a three-stage sample design in most urban areas. In rural areas, the villages were selected at the first stage using a Probability Proportional to Size (PPS) sampling scheme. The required number of households was selected at the second stage using systematic sampling. In urban areas, blocks were selected at the first stage, census enumeration blocks (CEB) containing approximately 150–200 households were selected at the second stage, and the required number of households were selected at the third stage using a systematic sampling technique. NFHS-3 collected information from 109,041 households and 124,385 women aged 15–49 years. The details of the survey design and implementation are given in the NFHS 2005–06 report [24].

This analysis is based on most recent singleton births to women from the rural northern region of India during the five years preceding the NFHS 2005–06. The northern region includes Rajasthan, Uttarakhand, Uttar Pradesh, Madhya Pradesh, Chattisgarh, Bihar and Jharkhand. This is due to the fact that data on antenatal care in NFHS 2005–06 were collected only for the most recent birth. Since NFHS 2005–06 uses a multistage sampling design, sampling weights are required to make the estimates representative. We used appropriate sampling weights for generating bivariate results presented in the paper. The details of the sampling weight can be obtained from the NFHS 2005–06 report [24].

The NFHS 2005–06 can be obtained free of cost for research purposes from the International Institute for Population Sciences Mumbai on request. This dataset can also be obtained from *MEASURE DHS* on request. The details about accessing data from *MEASURE DHS* are available on their website (http://www.measuredhs.com/data/Access-Instructions.cfm). For this analysis, we obtained the NFHS 2005–06 data from the International Institute for Population Sciences Mumbai.

### Outcome variable

The outcome variable of interest was neonatal mortality which is defined as the death of a live born baby within 28 days. The information on age at death for the live births taken place in five years preceding the NFHS 2005–06 was utilized to create the outcome variable.

### Exposure and control variables

The key exposure variables included any reported antenatal visit (ANC), iron and folic acid supplement (IFA) and doses of tetanus toxoid vaccinations (TT). Other variables controlled for in the discrete-time logistic regression model were skilled attendance at delivery, size of the newborn at birth (smaller than average, average, larger than average), sex of the neonate (male, female) and birth interval. The categories for birth interval were first birth order, second or third child and birth interval less than 24 months, second or third child and birth interval > = 24 months, fourth or higher child and birth interval <24 months, fourth or higher child and birth interval > = 24 months. Mother's age at birth (less than 20 years, 20–30 years, greater than 30 years), wealth quintiles (poorest, poorer, middle, richer, richest), religion (Hindu, Muslim, others), caste (Scheduled castes/tribes (SC/ST), other backward classes (OBC), others), and mother's education (non-literate, schooling upto middle school, middle school and higher) were also included in the models. Caste is a hereditary, endogamous, usually localised group, having a traditional association with an occupation, and a particular position in local hierarchy [25]. The scheduled castes/tribes and other backward classes are socially and economically less developed compared to the ‘other castes’. So, the Government of India has implemented a number of affirmative actions to improve the socio-economic status of these castes.

### Statistical analysis

We use multivariate discrete-time logistic model to examine the relationship between ANC use, IFA supplementation, and TT vaccination during pregnancy with the risk of neonatal mortality, after adjusting for other control variables. The outcome variable for the discrete-time logistic model was the binary indicator of occurrence of neonatal death. A number of recent studies have used this approach to model neonatal mortality [26]–[27]. As a model building strategy, we first fitted bivariate models for the association between each possible exposure/control variable and neonatal mortality. All the variables that were significant in the bivariate models (at a conservative *p*<.2) were included in the multivariable model. All the independent variables were tested for possible multi-collinearity before putting them into the discrete-time logistic regression model. The odds ratio and the 95% confidence interval (CI) for neonatal death were estimated for each factor. Having found a significant relationship between TT vaccination during pregnancy and risk of neonatal mortality, we further estimated the population attributable risk (PAR). The PAR is given by
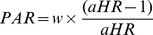
where w = proportion of women who did not receive > = 2 tt

aHR = adjusted hazard ratio for neonatal death in neonates born to women who did not receive > = 2 tt

Finally, we used binary logistic regression model to examine the factors associated with the utilization of two or more doses of TT vaccination during pregnancy. We used STATA 11.0 for statistical computations. All the independent variables were tested for possible multi-collinearity before putting them into the regression models.

## Results

In the five years preceding the survey round, there were 8,474 singleton most recent births. Of these 258 resulted in neonatal death. 59% of mothers reported receiving any form of antenatal care during their most recent pregnancy. 48% of mothers reported receiving iron and folic acid supplementation. Twenty-seven percent of mothers did not report receiving any tetanus toxoid vaccination during their most recent pregnancy. About two-thirds of mothers received two or more than two doses of tetanus toxoid (Table 1). Interestingly, only 25% of the recent births were supervised trained medical professionals. Approximately 15% of the recent births occurred to mothers who were less than 20 years of age. About 18% of births occurred to mothers who were above 30 years of age. Fifty-eight percent of births were of average size at birth and about 20% were of size larger than average.

**Table 1:** The prevalence of pregnancy health-care services provided to mothers and other birth related characteristics of infants born in five years preceding NFHS 3, rural northern India‡, 2005–06.

*Pregnancy health-care service and other birth related characteristics*	*%*	*N (weighted)*
**Antenatal care**		
No Antenatal care	41.3	3499
Availed antenatal care	58.7	4975
**Iron folic acid supplements**		
None	52.3	4434
Received	47.7	4043
**Tetanus toxoid vaccination**		
None	27.4	2320
1 injection	7.8	663
> = 2 injections	64.8	5495
**Type of supervision at birth**		
Untrained	74.8	6344
Trained	25.2	2136
**Mother's age at birth**		
<20 years	14.8	1253
20–30 years	67.0	5687
>30 years	18.2	1542
**Size of the newborn at birth**		
Smaller than average	22.6	1919
Average	57.6	4886
Larger than average	19.8	1675
**Birth interval**		
First birth	20.2	1713
2^nd^/3^rd^ birth and birth interval <24 months	10.2	870
2^nd^/3^rd^ birth and birth interval > = 24 months	27.9	2365
4^th^/higher birth and birth interval <24 months	9.4	795
4^th^/higher birth and birth interval > = 24 months	32.3	2739
**Total**	**100.0**	**8482**

Note:

‡ includes Rajasthan, Uttaranchal, Uttar Pradesh, Madhya Pradesh, Chattisgarh, Bihar, and Jharkhand.

The total number varies between categories because some values are missing.

Discrete-time logistic regression results adjusted for socio-economic, demographic, and health service delivery variables are presented in Table 2. Antenatal care provision and uptake of iron and folic supplements were not significantly associated with neonatal mortality. However, maternal tetanus vaccination was significantly and negatively associated with neonatal mortality: the odds of all-cause neonatal death were reduced following one or more antenatal dose of TT with odds ratios (OR) of 0.46 (95% CI 0.26 to 0.78) after one dose and 0.45 (95% CI 0.31 to 0.66) after two or more doses.

**Table 2:** Discrete-time logistic regression results for neonatal mortality, rural northern India‡, 2005–06.

*Pregnancy health-care service and other birth related characteristics*	*Odds ratio (95% CI)*	*p-value*
**Antenatal care**		
No Antenatal care (reference)		
Availed antenatal care	1.19 (0.83,1.73)	0.343
**Iron folic acid supplements**		
None (reference)		
Received	1.19 (0.86,1.64)	0.291
**Tetanus toxoid vaccination**		
None (reference)		
1 injection	0.46 (0.26,0.78)	0.005
> = 2 injections	0.45 (0.31,0.66)	0.000
**Type of supervision at birth**		
Untrained (reference)		
Trained	1.52 (1.13,2.03)	0.005
**Mother's age at birth**		
<20 years (reference)		
20–30 years	1.22 (0.83,1.80)	0.312
>30 years	1.47 (0.87,2.50)	0.153
**Size of the newborn at birth**		
Smaller than average (reference)		
Average	0.67 (0.50,0.88)	0.005
Larger than average	0.79 (0.55,1.14)	0.211
**Birth interval**		
First birth (reference)		
2^nd^/3^rd^ birth and birth interval <24 months	0.47 (0.28,0.77)	0.003
2^nd^/3^rd^ birth and birth interval > = 24 months	0.37 (0.25,0.55)	0.000
4^th^/higher birth and birth interval <24 months	0.95 (0.61,1.47)	0.814
4^th^/higher birth and birth interval > = 24 months	0.31 (0.20,0.48)	0.000

Note:

‡ includes Rajasthan, Uttaranchal, Uttar Pradesh, Madhya Pradesh, Chhattisgarh, Bihar, and Jharkhand.

CI – confidence interval.

Control variables included wealth quintiles, religion, caste, mother's education, and sex of the newborn.

Considering the plausible association between tetanus toxoid vaccination during pregnancy and neonatal mortality, we computed the population attributable risk (PAR). The PAR indicates that 16% of the neonatal deaths in rural northern India could be attributed to a lack of at least two doses of tetanus toxoid vaccination during pregnancy (PAR 0.16; 95% CI 0.05 to 0.27).

Type of supervision at birth was significantly associated with neonatal mortality in rural northern India. The odds of neonatal deaths were significantly higher among the deliveries supervised by trained professionals compared to those supervised by friends/relatives (OR: 1.52; 95% CI: 1.13–2.03). Second and third order births had significantly lower risk of neonatal mortality compared to the first order births. Likewise, babies of average size at the time of birth had significantly lower risk of neonatal mortality compared to babies that were smaller than average at the time of birth (OR: 0.67; 95% CI: 0.50–0.88). Interestingly, mother's age at the birth of the newborn was not associated with neonatal mortality when the results were adjusted for other exposure and control variables.

Finally, we examined factors associated with tetanus toxoid vaccination during pregnancy (Table 3). Mother's education, mother's age at birth of the index child and wealth status of the household were significantly associated with provision of at least two doses of antenatal tetanus vaccination, with educated and better-off mothers significantly more likely to receive at least two doses. Interestingly, mothers who were older than 20 years at the birth of index child were significantly less likely to receive vaccination (OR 0.78 and 0.50 for mothers aged 20–30 years and mothers aged more than 30 years, respectively). Muslim mothers were 0.85 (95% CI: 0.73, 0.98) times as likely as Hindu mothers to avail tetanus toxoid vaccination.

**Table 3:** Binary logistic regression analysis results for factors affecting utilization of two or more tetanus toxoid vaccinations, rural Northern India‡, 2005–06.

*Covariate & category*	*Odds ratio (95% CI)*	*p-value*
**Mother's education**		
Non-literate (reference)		
Schooling up to middle school	1.94 (1.71,2.20)	0.000
Middle school and higher	3.58 (2.79,4.58)	0.000
**Mother's age at birth of index child**		
Less than 20 years (reference)		
20–30 years	0.78 (0.68,0.90)	0.001
Greater than 30 years	0.50 (0.42,0.59)	0.000
**Caste**		
Scheduled castes/tribes (reference)		
Other backward classes	1.29 (1.16,1.44)	0.000
Others	0.95 (0.81,1.12)	0.564
**Religion**		
Hindu (reference)		
Muslim	0.85 (0.73,0.98)	0.031
Others	0.83 (0.61,1.13)	0.237
**Wealth quintiles**		
Poorest (reference)		
Poorer	1.40 (1.25,1.57)	0.000
Middle	1.68 (1.46,1.94)	0.000
Richer	2.49 (2.04,3.04)	0.000
Richest	5.95 (3.83,9.26)	0.000
**Type of household**		
Nuclear (reference)		
Non-nuclear	1.06 (0.96,1.17)	0.244

‡ includes Rajasthan, Uttaranchal, Uttar Pradesh, Madhya Pradesh, Chattisgarh, Bihar, and Jharkhand.

CI – confidence interval.

## Discussion and Conclusions

Results adjusted for socio-economic, demographic and health system related variables such as antenatal care and iron folic supplementation show a substantial protective impact of antenatal vaccination on neonatal mortality. The present findings are consistent with observational data from demographic surveillance settings [28] and those from a study that examined the determinants of neonatal mortality in a similar grouping of Indian states using a combination of sample registration and population survey data, where neonatal mortality was lower by 30% for children of mothers who received at least two doses of tetanus toxoid during pregnancy [29]. Our findings suggest that 16% of the neonatal deaths in rural northern India can be attributed to lack of at least two doses of tetanus toxoid vaccination during pregnancy.

The present findings should be interpreted in the context of stagnation of the decline in infant mortality in India in the recent past at unacceptably high levels for most of the states in the country [30] but especially in the Northern states. Policy initiatives have included *Janani Suraksha Yojana*, where women are paid cash incentives for delivering their babies in public health facilities or government-designated private facilities [31]. Delivery in a health facility is likely to contribute substantially to newborn survival (including tetanus prevention) through provision of hygienic delivery conditions and the ability to provide immediate newborn care. While the Government of India has been promoting antenatal care under the Reproductive and Child Health (RCH) Programme and the National Rural Health Mission [32], these programmes have several components that require the attention of health workers and it is possible that the crucial importance of achieving universal antenatal tetanus vaccination has not received sufficient emphasis.

The present findings are consistent with the presence of a substantial protective effect of a single antenatal dose of tetanus toxoid. The PAR indicates that 20% of the neonatal deaths in rural northern India could be attributed to a lack of at least one dose of tetanus toxoid vaccination during pregnancy (PAR 0.20; 95% CI 0.11 to 0.29). Two or more doses are generally recommended in order to assure high protective antibody titres. The unexpectedly substantial protective effect of a single dose requires explanation. It is possible that public health interventions including childhood vaccination of mothers through the EPI programme implemented by the WHO as early as 1970s' and reproductive health programmes that include tetanus vaccination of women in the reproductive age group have led to a higher proportion of north Indian women who have had prior vaccination and hence respond more vigorously to what is effectively a ‘booster’ single dose of vaccine given antenatally. Alternatively, there may have been advances in vaccine manufacture, storage and distribution logistics that have resulted in a higher immunological response. Further studies are needed to elucidate the biological basis of the present findings.

Interestingly, the supervised births had higher risk of neonatal mortality compared to the unsupervised births in rural northern India. The plausible reason for this type of finding is that in rural northern India, it is only the risky births or deliveries with complications that tend to take place under supervision of medically qualified professional. This is also obvious from the finding that only 25% of the recent births were delivered under the supervision of a medically qualified professional in rural northern India. Some other Indian studies have also reported similar findings [33].

The socio-economic component of the modelling indicates that poor, older, and Muslim women are significantly less likely to receive the recommended doses of tetanus toxoid vaccination. This is consistent with the findings of other studies that also document that older women are less likely than younger women to utilize antenatal and delivery care in the country [34]–[36]. These are also the women who are less likely to make use of other services such as skilled attendance at delivery, and schemes like Janani Suraksha Yojana [24], [31], [37]. Although, the National Rural Health Mission has a mandate to make quality health care accessible to all, particularly to the poor and the marginalized, our findings along with others clearly show that it is the poor and the marginalized who are still out of the reach of government-sponsored public health programmes [32]. These are the groups that bear the maximum burden of neonatal and infant mortality in the country. From the present findings, a policy and programming imperative to assure provision of at least a single antenatal dose to all, especially those in marginalised or disadvantaged population subgroups, is likely to achieve substantial impact on improving newborn survival. Relative to the complexities of providing the full health system functionality required for comprehensive and safe maternal and newborn care, this specific intervention should be within the reach of the public health system as an immediate priority.

The large sample size allowed the examination of health-care interventions and neonatal mortality, after adjusting for a range of other variables known to be associated with neonatal mortality. The focus on most recent singleton births in the analysis helped us to avoid recall bias on the part of mothers included in the study. However, limitations of the present study must be acknowledged. As the protective effects of tetanus last long – more than three-quarters of women will maintain ‘protective levels’ for 3 years [3], [38], an analysis based only on first births would have yielded more information. We could not base our multivariate analysis only on first births due to the small sample size and relatively small numbers of neonatal deaths. Secondly we could not validate the information provided by the women for example by cross reference to clinic records or biomarkers such as tetanus antibody levels. However it should be noted that the survey format has been regularly used in the various rounds of NFHS and the findings are widely used to monitor the performance of policies and programmes in India [39]. Survey teams receive formal training and quality control measures are in place. Also, findings from other similar large-scale surveys in India such as the District Level Household Surveys (DLHS) [37] – provide some cross reference to the credibility of information provided in NFHS 3. Thirdly, we could not estimate the extent to which the childhood vaccination of mothers (especially the younger ones) against six vaccine preventable diseases could have resulted into overestimation of the potential impact of one dose of tetanus toxoid vaccination during pregnancy. This is because the information on childhood vaccinations was not available for the mothers of the neonates. In cross-sectional surveys, one cannot rule out the possibility of underreporting of maternal tetanus toxoid vaccination by mothers whose infants had died during the neonatal period. However, this possibility is minimal in NFHS 2005–06 because the information on neonatal mortality was collected in the birth history section, whereas the information on maternal tetanus toxoid vaccinations was collected in the antenatal care section. It is also important to note that these two sections were well spaced out in the interview schedule.

Using population estimates from the ‘Population Projection for India and States 2001–2026’[40] and the population attributable risk estimated in the present study, we estimate that 78,632 (confidence bound: 24,573–132,692) neonatal deaths could be prevented in rural northern India every year by universal provision of at least two antenatal tetanus toxoid vaccination. Our findings, therefore, call for greater focus on increasing access to tetanus toxoid in rural northern India within a context of greater attention on the poor and the marginalized if India is to achieve the Millennium Development Goal 4 [41].
